# The Relationship between Metabolic Syndrome and Smoking and Alcohol Experiences in Adolescents from Low-Income Households

**DOI:** 10.3390/children8090812

**Published:** 2021-09-16

**Authors:** Moonyoung Choi, Joungkyue Han, Yonghwan Kim, Jinwook Chung

**Affiliations:** 1 Department of Sports Science Convergence, Dongguk University, Seoul 04620, Korea; dory0301@dongguk.edu; 2 College of Sports Science, Chung-ang University, Anseong 17546, Korea; jkhan@cau.ac.kr; 3 Department of Physical Education, Gangneung-Wonju National University, Gangneung 25457, Korea; yhkim@gwnu.ac.kr

**Keywords:** metabolic syndrome, adolescent, health behaviors, smoking exposure, alcohol consumption, hand grip strength, physical activity, low income household

## Abstract

Metabolic syndrome (MetS) in children and adolescents is increasing globally and the age of onset is gradually decreasing. MetS is associated with serious health problems and presents an early risk for adult morbidity and mortality. From 2014–2019, we investigated the relationship between MetS and health behaviors such as smoking, alcohol consumption, and nutrition education in Korean adolescents (boys: 1235, girls: 1087, age: 13–18 years) based on household income; the relationship with hand grip strength was also evaluated. The prevalence of MetS was 8.8% in boys and 5.1% in girls; in the lowest income households, the risk increased ~1.5-fold for boys and ~4-fold for girls, whereas risks of smoking and alcohol use increased 1.81 vs. 2.34 times, and 2.34 vs. 2.37 times for boys and girls, respectively. In adolescents with the weakest grip strength, the risk of MetS increased 9.62 and 7.79 times in boys and girls, respectively. Girls lacking nutrition education exhibited a 1.67-fold increased risk of MetS, but this was not significant in boys. Low household income increased the risk of unhealthy behaviors such as smoking and alcohol consumption in both sexes, and together with low hand grip strength, was an important predictor for developing MetS.

## 1. Introduction

Metabolic syndrome (MetS) is defined as a cluster of factors that increase the risk for cardiovascular disease (CVD) and diabetes, including increased waist circumference, high systolic blood pressure, high triglyceride (TG) levels, elevated fasting blood sugar, and low high-density lipoprotein cholesterol (HDL-C) levels [1,2,3]. The overall prevalence of MetS is 22–44% [4]. In addition to the increase in obesity worldwide, the prevalence of MetS in children and adolescents is increasing [5]. The prevalence of MetS in children and adolescents in the total population is known to be 3.3%, and it has been reported that the prevalence of obesity in adolescents has increased to 29.2% [6]. Childhood MetS contributes to serious health problems and is an early risk factor for considerable adult morbidity and mortality [7]. The increased prevalence of MetS is mainly associated with abdominal obesity and increased sedentary lifestyles [8]. Therefore, in the field of public health, much attention is focused on health behavior modification to influence lifestyle changes in the general public in order to reduce obesity and increase physical activity [9]. Health behaviors are habits that affect the health of individuals. This includes behaviors that promote health, such as physical activity and proper nutrition, as well as behaviors that increase the risk of disease, such as smoking and alcohol consumption [10]. Many of the leading causes of death and illness result from poor health behaviors. Unhealthy behaviors further increase the risk of MetS [11], and the more unhealthy behaviors in childhood, the higher the MetS in adulthood is predicted [12]. In particular, among health behaviors, physical activity is a factor that is greatly affected by the family physical activity environment in children and adolescents [13]. In addition, physical activity levels should be considered more specifically, as they can influence the way blood glucose is processed [14]. Previous studies have reported that lack of physical activity is associated with lower hand grip strength (HGS) [15,16,17]. Low HGS is a risk indicator for MetS in adults, and a similar risk is exhibited in adolescents [18]. A recent study reported a nonlinear relationship between HGS and the prevalence of MetS in adolescents [19]. HGS is correlated with low muscle mass and total strength, and HGS testing is a simple and safe measure that can predict many risks, including diabetes, CVD, and mortality [20,21]. Studies have shown that adult physical activity level and HGS are related to household income [22,23,24], and it was shown that parental socioeconomic status impacts children’s health behaviors, such as physical activity, smoking and alcohol [25,26,27,28]. In addition, there is considerable evidence supporting an inverse relationship between socioeconomic status and prevalence of MetS. It is known that the prevalence of MetS is significantly higher in households with the lowest household incomes compared to households with average and high household incomes [29,30]. Adolescents from socio-economically disadvantaged environments are more likely to engage in unhealthy behaviors, which is thought to increase the prevalence of MetS, but previous studies alone have limitations in explaining the relationship between them. Reducing the risk of MetS in adolescents from low-income households requires an improved public awareness that health behavior modification should be a major focus. Therefore, this study investigated the effect of the relationship between household income, HGS and health behavior on the prevalence of MetS in Korean adolescents. It hypothesized that adolescents from low income households would exhibit a higher prevalence of MetS and a higher risk of engaging in unhealthy behaviors such as smoking and alcohol consumption than adolescents with high household income.

## 2. Materials and Methods

### 2.1. Participants

**Figure 1:**
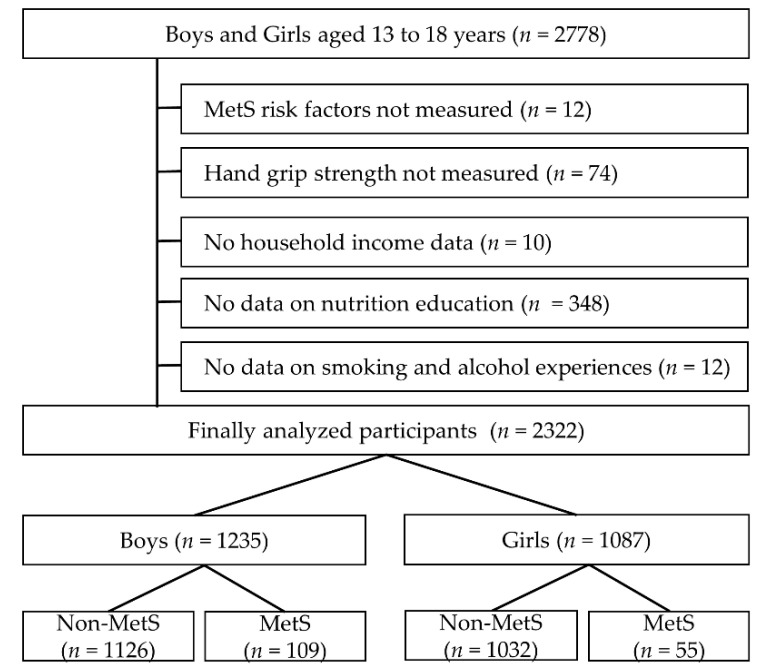
Participant’s inclusion and exclusion process.

To investigate the relationship between health behaviors related to household income and the prevalence of MetS, 2322 adolescents (boys: 1235, girls: 1087) aged 13–18 years who participated in the Korea National Health and Nutrition Survey (KNHNS) from 2014 to 2019 were included in this study. Initially, a total of 2778 adolescents consented and participated to provide data for research purposes during the study period. However, those who did not complete the MetS risk factor measurement (*n* = 12), had no HGS measurement (*n* = 74), had no income information (*n* = 10), did not provide information on smoking and alcohol (*n* = 12), or did not provide information on nutrition education (*n* = 348) were excluded from the study (Figure 1). In this study, in order to comply with research ethics, the purpose of the research and the purpose of the examination of the results were explained to adolescents and legal guardians, and written informed consent was obtained. In addition, it was approved by the research ethics committee of the affiliated institution, the Korean Disease Control and Prevention Agency (2015-01-02-6C, 2 January 2015) and Gangneung-Wonju national university (R2020-16, 18 March 2020).

### 2.2. Metabolic Syndrome

In this study, the criteria proposed by Cook et al. were used to diagnose MetS in adolescents [31]. Since the criteria for MetS in adults have not been formally defined or applied to children or adolescents, Cook et al. modified the adult criteria to the closest representative values available from pediatric reference data to diagnose MetS in adolescents. MetS was defined as the presence of three or more of the following components: waist circumference ≥ 90th percentile, blood pressure (BP) ≥ 90th percentile, HDL-C ≤ 40 mg/dL, fasting blood glucose ≥ 110 mg/dL, and TG ≥ 110 mg/dL. Additionally, having a history of drug treatment for any of these components was defined as possessing that component.

### 2.3. Hand Grip Strength

An individual’s HGS level is highly correlated with the level of physical activity. Previous studies reported that lower HGS is associated with lower levels of physical activity and lower muscle mass. In this study, HGS data were analyzed to assess the participant’s physical activity level [22]. HGS was measured using a digital dynamometer (TKK 5401, TAKEI, Niigata, Japan). Participants assumed a standing position with backs straight while looking straight ahead with legs shoulder-width apart, and both feet facing forward. The arms were allowed to hang naturally, ensuring that the elbows or wrists were not bent, with the arms not touching the torso. Also, care was taken to maintain the basic posture during measurement of the HGS. The grip of the dynamometer was adjusted so that the second joint of the participant’s index finger was at 90° [32]. The maximum HGS was measured three times each on the left and right by crossing both hands, and a rest period of 60 s was provided between the measurements. For the analysis, the data of the hand with the highest absolute value measured among both hands was used. The value obtained by dividing the highest measured absolute value by the body weight was normalized to a percentage and used as a relative value. The measured grip strength was graded using quartiles for analysis, with the strongest group classified as G1 and the weakest group as G4.

### 2.4. Household Income and Health Behaviors

The socioeconomic characteristics were determined using an interview survey, and the monthly income of the parents was used to define the household income of the adolescents. Measured household income was graded with G1 for the highest income group and G5 for the lowest income group using the quintile for analysis. Health behaviors with regard to smoking, alcohol consumption, and nutrition were surveyed using self-reported questionnaires with “yes” or “no” responses.

### 2.5. Data Analysis

SPSS 25.0 (SPSS Inc., Chicago, IL, USA) was used for data analysis. The Shapiro–Wilk test for normality was performed and the main variables for analysis did not exhibit a normal distribution (*p* < 0.05). Therefore, a non-parametric statistical method was applied to compare the general characteristics of the sex analysis (Table 1) and the MetS and the non-MetS groups (Table 2). Among the general characteristics in Table 2, continuous variables were expressed as means and standard deviations, and a non-parametric Mann–Whitney test was used. Income quintiles, HGS quartiles, and categorical variables such as nutrition education, smoking and alcohol use (Table 3) were recorded as percentages, and the chi-square test was performed. For the prevalence of MetS, the prevalence of MetS risk factors, and smoking and alcohol exposure based on household income level, logistic regression analysis was performed to calculate the odds ratio (OR). The correction variables were crossed with age, household income, HGS, nutrition education, and smoking and alcohol experiences. The significance level was set to *p* < 0.05, and the confidence interval (CI) of the odds ratio was set to 95%.

## 3. Results

### 3.1. General Characteristics of Participants

Participants were classified based on sex, and the general characteristics are shown in Table 1. There was no significant difference in age when boys and girls were compared, but there were significant differences in height, weight, and body mass index (BMI).

### 3.2. MetS Prevalence According to Risk Factors, Household Income, HGS and Health Behaviors

MetS was diagnosed in 109 (8.8%) of 1126 boys and 55 (5.1%) of 1032 girls. When the risk factors for MetS were compared between the non-MetS and MetS groups, there were significant differences in waist circumference, systolic blood pressure (SBP), diastolic blood pressure (DBP), TG, fasting blood glucose, and HDL-C for both boys and girls. There was no significant difference between the non-MetS and MetS groups regarding the household income of boys, but there was a significant difference for the girls (*p* = 0.039). Relative HGS was significantly different between non-MetS and MetS groups for both boys (*p* < 0.001) and girls (*p* < 0.001). Health behavior factors included nutrition education, smoking and alcohol consumption. Regarding nutrition education, there was no significant difference in boys, but there was a significant difference between non-MetS (21.9%) and MetS (17.5%) groups in girls (*p* = 0.028). There was no significant difference in smoking and alcohol consumption for both boys and girls (Table 2).

### 3.3. MetS Odds Ratio According to Household Income, HGS and Health Behaviors

Household income was analyzed by its classification into quintiles with G1 representing the highest group and G5 the lowest. In the group with the lowest household income, the risk of developing MetS increased 1.45 times (*p* = 0.041) for boys and 4.05 times (*p* = 0.018) for girls compared to the group with the highest household income. HGS was analyzed by grouping it into quartiles. G1 represented the strongest HGS group, and G4 the weakest. In the group with the weakest HGS, the risk of developing MetS increased 9.62 times (*p* < 0.001) for boys and 7.79 times (*p* < 0.001) for girls. Among health behavior factors, nutritional education increased the risk of developing MetS 1.67 times in girls without education compared to girls with education (*p* = 0.043), but did not increase significantly in boys. Smoking and alcohol did not significantly increase the risk of developing MetS in exposed adolescents compared to non-exposed adolescents (Table 3).

### 3.4. Relationship between Smoking and Alcohol Experience, and MetS Risk Factors

In the case of smoking exposure, there were significant differences in waist circumference (*p* = 0.043), SBP (*p* = 0.041) and DBP (*p* < 0.001) in boys, but there were no significant differences in TG, fasting blood glucose, and HDL-C. In girls, there was a significant difference only in DBP (*p* = 0.010) based on smoking exposure. In the case of alcohol consumption, there were significant differences in waist circumference (*p* = 0.001), SBP (*p* = 0.012), DBP (*p* = 0.001), and TG (*p* = 0.043) in boys, but there was no significant difference in fasting blood glucose and HDL-C. In girls, there was a significant difference only in waist circumference (*p* = 0.022) according to alcohol consumption (Table 4).

### 3.5. Smoking and Alcohol Consumption Odds Ratios according to Household Income

In the group with the lowest household income, the risk of experiencing smoking increased by 1.81 times (*p* = 0.020) for boys and 2.34 times (*p* = 0.012) for girls compared to the group with the highest household income. The risk of alcohol consumption increased 2.34 times (*p* = 0.012) for boys and 2.37 times (*p* < 0.001) for girls in the group with the lowest household income compared to the group with the highest household income (Table 5).

## 4. Discussion

The prevalence and risk factors of MetS are affected by individual psychosocial factors [33,34] and health behaviors such as exercise, nutrition, alcohol intake and smoking [35,36,37]. The combination of poor health behaviors such as lack of physical activity, poor diet, smoking, and excessive alcohol consumption with factors such as frequent stress and low socioeconomic status greatly increases the prevalence of MetS. Recent studies have reported that socioeconomic factors such as household income are related to the prevalence of MetS and CVD [38]. In addition, low household income is strongly associated with adolescents’ level of health behavior awareness, which may have a significant impact on increased prevalence of MetS [39].

The mechanism by which low household income influences health behavior cannot be fully explained by the issue of simply being unable to purchase health-promoting goods and services. Logically, smoking and consuming alcohol are behaviors that expend resources on products that are unhealthy, whereas an exercise such as walking is a behavior that requires no expenditure. Ironically, it is known that individuals with lower household incomes spend more on unhealthy behaviors and participate less in health-promoting behaviors that cost little [9]. Previous studies investigated the relationship between household income and health behavior to explain why these contrary health behaviors are observed in individuals with low household incomes. According to a study by Lantz et al., people with lower household incomes were more likely to seek aid in controlling their mood through smoking, overeating, drinking and inactivity when faced with stressful situations [40]. Siahpush et al. reported a lack of knowledge and access to information on how health behavior affects health risks as another reason [41]. Individuals with fewer learning opportunities or lower educational attainment due to unfavorable socioeconomic circumstances may be less motivated to adopt healthy behaviors as their knowledge of the risks of unhealthy behaviors may be limited. This study attempted to determine whether household income is a factor influencing the prevalence of MetS and health behavioral factors such as smoking, alcohol, nutrition education experience, and physical activity in adolescents as well.

In the results of this study, the prevalence of MetS in adolescents based on household income differed according to sex, and only girls exhibited a significant inverse relationship. However, the risk of developing MetS for both boys and girls showed a tendency to increase with lower household income. These results imply that all adolescents who are placed in an unfavorable socioeconomic environment are exposed to an increased risk of MetS, regardless of sex.

HGS is correlated with total strength and muscle mass related to physical activity among health behaviors, and weak HGS indicates a lack of physical activity [20,21]. In addition, weak HGS is a clinical indicator of stamina deterioration, as it has been associated with many negative health outcomes [42]. As expected, low HGS in this study was a strong predictor of MetS in both boys and girls. In adolescents, the risk of developing MetS significantly increased as the HGS decreased.

Balanced nutrition prevents many diseases and plays an important role in improving physical and intellectual efficiency [36]. In particular, an unfavorable socioeconomic environment is considered as one of the causes of poor nutrition. In the results of this study, the prevalence and risk of MetS were significantly increased only in girls without nutrition education. These results are thought to be due to the fact that adolescent girls are more sensitive to changes in body weight and body shape according to diet than boys and have higher adherence to nutrition education.

For both smoking and alcohol consumption, the risk of exposure significantly increased with lower household income for both boys and girls. Although the prevalence and risk of MetS did not significantly increase in the smoking and alcohol consuming boys, the risk factors for MetS such as waist circumference, SBP, DBP and TG were significantly higher. Also, in the girls, DBP was significantly higher when they smoked, and waist circumference was significantly higher when they consumed alcohol. These results are partly consistent with the results of a study by Slagter et al. [37] who reported that smoking and alcohol were highly correlated with an increased risk of abdominal obesity and hypertension. Previous studies have reported a significant decrease in HDL-C levels with higher smoking frequency and alcohol consumption. However, this study did not find any concordant results by analyzing experiences with only “yes” or “no” responses. The components of MetS, such as low HDL-C, high BP and fasting blood glucose, and abdominal obesity, are each considered a disease. However, even if the number is at the borderline level, it is necessary to carefully consider each risk factor because several of them form a cluster and contribute to an increase in the risk of CVD and diabetes.

In this study, adolescents who were placed in socioeconomically disadvantaged environments such as low household income had weak muscle strength and less health-related knowledge such as nutrition education. This was also demonstrated to significantly increase the likelihood of exposure to the risk of engaging in smoking or drinking alcohol. This suggests that adolescents in low-income households are more likely to experience adverse social, physical and economic environments that may contribute to worse health outcomes. Consequently, adolescents from low-income households are more likely to develop MetS because they lack access to information promoting or harming health and are at higher risk of experiencing unhealthy behaviors. These results indicate that public policy interventions focused on mitigating the adverse effects of low household income on health behavior are required because socioeconomic disparity is a problem that cannot be effectively overcome solely by individual efforts.

This study has several limitations. First, it was not possible to filter out false reports as the analysis was based on data recorded in the self-report questionnaire. Second, quantitative data such as the amount or frequency of smoking and alcohol consumption were not obtained. Third, it was not possible to distinguish between those who had discontinued alcohol use and those who were current drinkers. Finally, the physical strength measurement method for estimating lack of physical activity was limited to HGS. Although previous studies reported that higher cardiorespiratory health is associated with lower MetS prevalence, this study did not measure aerobic exercise capacity due to the environmental limitations of a large-scale investigation [43]. Further studies are required to address these limitations.

Notwithstanding these limitations, this study has the strengths of providing significant information on the relationship between household income and health behavior as a predictor of MetS in adolescents. This information can be helpful in improving public awareness that modifying health behavior in order to reduce the risk of MetS in adolescents from low-income households should be a priority.

## 5. Conclusions

In this study, low household income increased the risk of unhealthy behaviors such as smoking and alcohol consumption in both boys and girls, and was an important determinant of MetS risk along with low hand grip strength. These findings suggest that household income is another predictor of the prevalence of MetS in adolescents. Therefore, social interventions to prevent MetS among adolescents who are placed in an unfavorable socioeconomic environment is important, and efforts are needed to systematize national health education programs so that all adolescents can equally establish awareness of health-promoting behaviors.

## Figures and Tables

**Table 1:** General characteristics of participants.

	Boys (*n* = 1235)	Girls (*n* = 1087)	*p*
Age, years	15.4 ± 1.7	15.5 ± 1.7	0.359
Height, cm	171.2 ± 7.0	160.5 ± 5.5	<0.001 *
Weight, kg	64.8 ± 13.9	55.1 ± 10.6	<0.001 *
BMI, kg/m^2^	22 ± 4.1	21.3 ± 3.7	<0.001 *

* *p* < 0.05; The values are shown as the mean ± standard deviation; BMI, body mass index.

**Table 2:** General characteristics according to the prevalence of metabolic syndrome.

	Boys			Girls		
	Non-MetS	MetS	*p*	Non-MetS	MetS	*p*
*n* (%)	1126 (91.2%)	109 (8.8%)		1032 (94.9%)	55 (5.1%)	
Age, years	15.4 ± 1.8	15.5 ± 1.7	0.603	15.4 ± 1.7	15.7 ± 1.8	0.226
Height, cm	171.0 ± 7.0	173.7 ± 6.5	<0.001 *	160.5 ± 5.4	162.2 ± 6.5	0.023
Weight, kg	62.9 ± 12.1	85.6 ± 13.3	<0.001 *	54.0 ± 9.2	74.0 ± 15.4	<0.001 *
BMI, kg/m^2^	21.4 ± 3.6	28.3 ± 3.8	<0.001 *	21.0 ± 3.2	28.1 ± 5.5	<0.001 *
MetS risk factors						
Waist circumference, cm	73.8 ± 9.5	92.9 ± 9.5	<0.001 *	68.6 ± 7.3	85.2 ± 12.2	<0.001 *
SBP, mmHg	111.2 ± 9.1	124.8 ± 8.9	<0.001 *	105.3 ± 8.3	117.6 ± 10.5	<0.001 *
DBP, mmHg	67.3 ± 8.6	76.3 ± 9.6	<0.001 *	66.8 ± 7.5	75.9 ± 7.8	<0.001 *
TG, mg/dL	81.3 ± 47.4	152.8 ± 67.4	<0.001 *	80.5 ± 39.3	153.9 ± 62.3	<0.001 *
Glucose, mg/dL	92.1 ± 7.3	96.5 ± 18.4	0.015 *	89.2 ± 6.8	97 ± 12.7	<0.001 *
HDL-C, mg/dL	50.0 ± 9.0	39.7 ± 6.4	<0.001 *	53.9 ± 9.8	43.1 ± 8.3	<0.001 *
Family income, Korean Won	493.9 ± 293.8	523 ± 316.9	0.328	506.9 ± 308.5	426.6 ± 273.1	0.039 *
Grip strength, kg/BW, %	53.8 ± 11.2	42.7 ± 10.2	<0.001 *	41.9 ± 8.2	33.5 ± 8.7	<0.001 *
Nutrition education, Yes, %	21.9	17.5	0.318	24.2	14.6	0.028 *
Smoking experience, Yes, %	18.1	16.5	0.674	7.6	5.5	0.563
Alcohol experience, Yes, %	36.9	36.7	0.968	29.4	30.9	0.806

* *p* < 0.05; The values are shown as the mean ± standard deviation or percent; BMI, body mass index; MetS, metabolic syndrome; SBP, systolic blood pressure; DBP, diastolic blood pressure; TG, triglycerides; HDL-C, high-density lipoprotein cholesterol.

**Table 3:** Metabolic syndrome odds ratio according to household income, grip strength and health behaviors.

	Boys			Girls		
	OR	95% CI	*p*	OR	95% CI	*p*
Family income						
G1	Reference	-	-	Reference	-	-
G2	0.774	0.401–1.493	0.445	2.585	0.926–7.216	0.070
G3	0.911	0.410–2.025	0.819	2.833	0.994–8.074	0.051
G4	1.025	0.579–1.812	0.933	3.590	1.257–10.253	0.017 *
G5	1.450	1.030–2.933	0.041 *	4.050	1.326–12.369	0.014 *
Grip strength						
G1	Reference	-	-	Reference	-	-
G2	3.466	1.115–7.769	0.032	0.787	0.209–2.965	0.724
G3	8.568	2.972–14.699	<0.001 *	2.227	0.762–6.504	0.143
G4	9.622	4.345–18.901	<0.001 *	7.792	3.002–13.223	<0.001 *
Nutrition education						
Yes	Reference	-	-	Reference	-	-
No	1.543	0.667–3.573	0.311	1.668	1.036–2.974	0.043 *
Smoking experience						
No	Reference	-	-	Reference	-	-
Yes	1.035	0.519–2.067	0.922	1.135	0.302–4.264	0.851
Alcohol experience						
No	Reference	-	-	Reference	-	-
Yes	1.184	0.710–1.974	0.518	1.029	0.501–2.113	0.938

* *p* < 0.05. OR, odds ratio; CI, confidence interval.

**Table 4:** Relationship between smoking and alcohol experience and risk metabolic syndrome factors.

	Non-Smoking	Smoking	*p*	Non-Alcohol	Alcohol	*p*
Boys						
Waist circumference, cm	75.2 ± 10.8	76.7 ± 11.3	0.043 *	74.7 ± 10.7	76.8 ± 11.2	0.001 *
SBP, mmHg	112.1 ± 9.7	114.4 ± 10.5	0.041 *	111.8 ± 9.8	113.3 ± 10	0.012 *
DBP, mmHg	67.7 ± 9.1	70.1 ± 8.7	<0.001 *	67.1 ± 9.2	69.9 ± 8.7	0.001 *
TG, mg/dL	87.3 ± 54.5	87.6 ± 46.5	0.943	85.7 ± 50.3	90.3 ± 57.5	0.043 *
Glucose, mg/dL	92.6 ± 7.4	92.3 ± 14.0	0.640	92.8 ± 7.6	92.0 ± 11.0	0.134
HDL-C, mg/dL	49.0 ± 9.2	49.5 ± 9.8	0.465	49.1 ± 9.1	49.0 ± 9.5	0.836
Girls						
Waist circumference, cm	69.4 ± 8.4	70.1 ± 9.3	0.528	69.1 ± 8.3	70.4 ± 8.8	0.022 *
SBP, mmHg	106.0 ± 8.9	105.1 ± 8.2	0.340	106.0 ± 8.9	105.9 ± 8.9	0.818
DBP, mmHg	67.1 ± 7.9	69.4 ± 6.6	0.010 *	67.0 ± 7.9	67.7 ± 7.5	0.235
TG, mg/dL	84.4 ± 43.8	83.5 ± 44.6	0.856	84.0 ± 41.1	85.2 ± 49.8	0.669
Glucose, mg/dL	89.7 ± 7.5	88.5 ± 6.0	0.098	89.8 ± 7.5	89.1 ± 7.1	0.112
HDL-C, mg/dL	53.4 ± 9.9	53.0 ± 10.6	0.767	53.5 ± 10.0	53.1 ± 10.0	0.602

* *p* < 0.05. The values are shown as the mean ± standard deviation or percent (%). SBP, systolic blood pressure; DBP, diastolic blood pressure; TG, triglycerides; HDL-C, high-density lipoprotein cholesterol.

**Table 5:** Smoking and alcohol experience odds ratios according to household income.

	Smoking Experience			Alcohol Experience		
	OR	95% CI	*p*	OR	95% CI	*p*
boys						
G1 (highest)	Reference	-	-	Reference	-	-
G2	0.758	0.494–1.163	0.204	0.837	0.601–1.165	0.292
G3	0.692	0.438–1.094	0.115	0.776	0.547–1.101	0.155
G4	1.147	0.745–1.766	0.533	1.134	0.926–1.671	0.854
G5 (lowest)	1.807	1.097–2.976	0.020 *	1.351	1.073–2.090	0.017 *
Girls						
G1 (highest)	Reference	-	-	Reference	-	-
G2	0.697	0.326–1.488	0.350	1.068	0.693–1.646	0.765
G3	1.008	0.483–2.103	0.983	1.399	0.898–2.179	0.138
G4	1.466	0.652–3.299	0.355	2.111	1.326–3.360	0.002 *
G5 (lowest)	2.337	1.202–4.541	0.012 *	2.365	1.406–3.979	0.001 *

* *p* < 0.05. OR, odds ratio; CI, confidence interval.

## Data Availability

Publicly available datasets were analyzed in this study. This data can be found here: https://knhanes.cdc.go.kr (accessed on 15 August 2021).
